# Engineering Glioma-Cell-Derived Exosomes as Trojan Horse for Precisely Targeted Chemotherapy of Glioblastoma

**DOI:** 10.34133/bmr.0365

**Published:** 2026-06-02

**Authors:** Zhijun He, Zhe Meng, Guoxi Luan, Pengbo Li, Chunyi Yang, Yifan Liang, Xiaodan Sun, Lingyun Zhao, Guihuai Wang, Xiumei Wang

**Affiliations:** ^1^ State Key Laboratory of New Ceramic Materials, Key Laboratory of Advanced Materials, School of Materials Science and Engineering, Tsinghua University, Beijing 100084, China.; ^2^ Department of Neurosurgery, Beijing Tsinghua Changgung Hospital, School of Clinical Medicine, Tsinghua University, Beijing 102218, China.; ^3^ School of Life Science, Tsinghua University, Beijing 100084, China.; ^4^ Department of Neurosurgery, Shandong Provincial Third Hospital, Shandong University, Shandong 250031, China.; ^5^ Department of Neurology, Beijing Tsinghua Changgung Hospital, School of Clinical Medicine, Tsinghua University, Beijing 102218, China.

## Abstract

The management of glioblastoma multiforme (GBM) remains challenging due to its poor prognosis and extremely high postoperative recurrence rate. Although the Gliadel wafer locally delivers carmustine, its clinical application is limited by suboptimal therapeutic efficacy and poor conformability to irregular resection cavities. To overcome these limitations, we developed a novel drug delivery system based on thermosensitive injectable chitosan-β-glycerol phosphate hydrogel containing doxorubicin-loaded mesoporous silica nanoparticles encapsulated within tumor-derived exosomes (Exo-DMSNs@CS). In an orthotopic mouse GBM relapse model recapitulating clinical tumor resection, the liquid Exo-DMSNs@CS formulation was injected into the surgical cavity and subsequently gelated in situ, achieving seamless adhesion to the irregular resection margins of the cavity. The exosome-coated nanoparticles (Exo-DMSNs), released gradually from chitosan hydrogel, exhibited enhanced tumor-targeting capability via exosome-mediated tumor-homing performance, thereby substantially promoting drug internalization. Compared to non-exosomal controls (DMSNs@CS), Exo-DMSNs@CS markedly suppressed tumor recurrence and prolonged survival. Our findings demonstrate that this Trojan-horse-inspired delivery strategy—leveraging tumor-derived exosomes to encapsulate drug-loaded nanoparticles—enables a localized and precise tumor-targeted drug delivery system, representing a promising therapeutic paradigm for GBM treatment.

## Introduction

Glioblastoma multiforme (GBM), one of the most lethal malignancies worldwide, has no effective therapeutic options [1]. The inevitable postoperative tumor recurrence and limited therapeutic efficacy of chemoradiotherapy contribute to its extremely poor prognosis [2,3]. The 2- to 4-week therapeutic gap between tumor resection and the following adjuvant chemoradiotherapy creates a critical window for tumor recurrence [4].

To address this challenge, a biodegradable Gliadel wafer containing a chemotherapy drug was developed for localized delivery of carmustine directly into the resection cavity [5]. Compared to systemic chemotherapy drug administration, this localized approach can enhance drug concentration at the tumor site while minimizing systemic toxicity [6,7]. However, the rigid wafer design suffers from poor conformability to irregular resection cavities and provides limited clinical benefit to patients [5,8]. Therefore, these limitations underscore the urgent need for novel drug delivery systems with improved antirecurrence efficacy and better cavity adaptation. The passive diffusion of carmustine from the traditional Gliadel wafer is insufficient to prevent the recurrence of GBM. Moreover, drug development for GBM has stagnated in recent decades due to the tumor’s pronounced heterogeneity [5,9]. Targeted drug delivery approaches based on local implantation may overcome these therapeutic limitations. Importantly, such targeted strategies could potentially reduce the side effects associated with the high drug doses required for passive diffusion systems like Gliadel wafers [10,11].

Recent advances have demonstrated that tumor-derived cell membrane and exosome-coated drug-loaded nanoparticles can considerably enhance drug delivery efficiency and therapeutic benefit across various cancers, including breast cancer [12–14], colon carcinoma [15], melanoma [16], fibrosarcoma [17], and glioblastoma [18,19], when administered systemically [20]. The homotypic targeting capability of these biomimetic nanoparticles stems from their preserved membrane proteins, which promote preferential uptake by their cell of origin [12,21]. Among these, tumor-derived exosomes, naturally secreted extracellular vesicles from cancer cells, exhibit superior tumor targeting, transmembrane transport, and immune evasion properties in drug delivery compared to liposomes or tumor cell membrane [22]. By combining these tumor-targeting advantages with enhanced intercellular uptake, tumor-derived exosome-mimetic drug-loaded nanoparticles could substantially improve the therapeutic efficacy of localized drug delivery for postresected GBM. However, systemic administration routes lead to substantial hepatic sequestration of most exosome-coated nanoparticles [12,16]. Thus, local delivery of exosome-coated nanoparticles emerges as a promising strategy to simultaneously improve tumor targeting while avoiding liver clearance [23]. Together, we designed a Trojan-horse-like delivery strategy by using a thermosensitive chitosan (CS)-based hydrogel delivery system to locally release tumor-derived exosome-coated drug-loaded nanoparticles for targeted GBM therapy.

Chitosan is a linear cationic polysaccharide derived from the deacetylation of chitin. Chitosan hydrogels possess excellent biocompatibility, biodegradability, tunable porosity, and responsiveness to environmental stimuli such as pH, temperature, and ionic strength, rendering them promising candidates for biomedical applications in drug delivery, wound healing, and tissue engineering. The intrinsic thermosensitivity of CS/β-glycerol phosphate (CS-GP) enables it to undergo gelation at body temperature to precisely conform to the irregular shape of tumor resection cavities. Meanwhile, the thermosensitivity guarantees maximally simplified surgical procedures and improved operational feasibility. Furthermore, the hydrogel provides suitable mechanical strength to protect vulnerable brain tissue from additional injury. Beyond the thermosensitivity of CS-GP, the positive charge carried by the chitosan enables efficient exosome loading and controlled release owing to the electrostatic interaction between the negative charge on exosomes and the positive charge on chitosan and the relatively slow degradation rate of chitosan. Given the high postoperation relapse rate of glioblastoma, a controllable release rate, achieved by adjusting the degradation rate of the hydrogel, can extend the postoperative therapeutic window and improve prognosis.

**Fig. 1.**
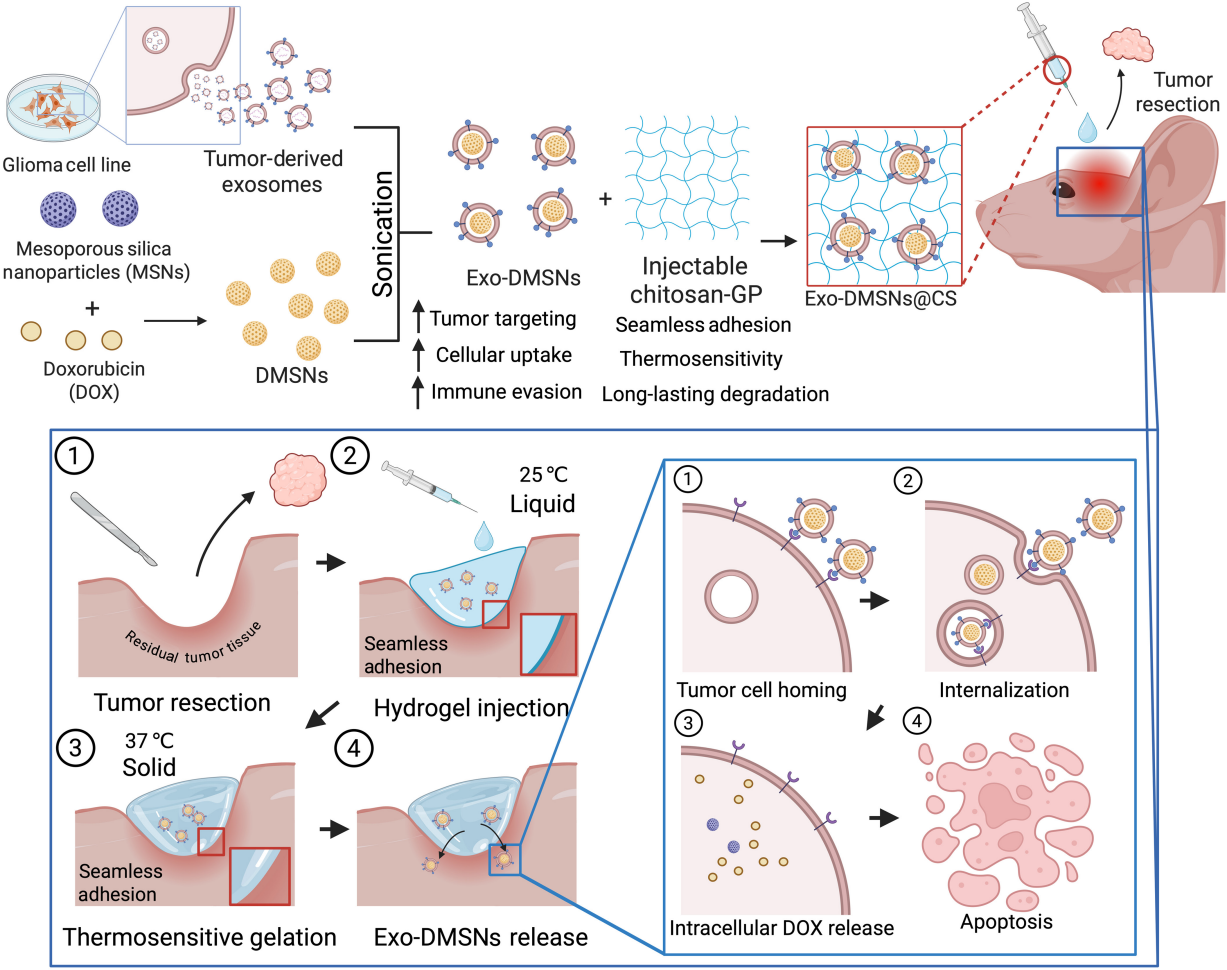
Schematic diagram illustrating the fabrication processes of the chitosan-β-glycerol phosphate hydrogel containing doxorubicin (DOX)-loaded mesoporous silica nanoparticles encapsulated within tumor-derived exosomes (Exo-DMSNs@CS) and application for the orthotopic glioblastoma multiforme (GBM) resection mouse model. The diagram was created via biorender.com.

In this study, we successfully fabricated doxorubicin (DOX)-loaded mesoporous silica nanoparticles (MSNs) that were encapsulated within tumor-derived exosomes (denoted as Exo-DMSNs). These Exo-DMSNs were subsequently incorporated into CS hydrogels for in situ injection. Upon implantation, the Exo-DMSNs released from the chitosan hydrogel were preferentially internalized by the tumor cells from which the exosomes were derived, rather than by normal brain cells, thereby helping prevent GBM recurrence while minimizing off-target side effects (Fig. 1). This localized implantation strategy overcomes the limited drug delivery due to the blood–brain barrier (BBB) and the rapid clearance of nanoparticles by the liver and kidney typically in intravenous administration, while also mitigating the systemic side effects [16,17,24]. To better simulate the clinical surgery treatment of GBM, an orthotopic GBM postresection mouse model was established to recapitulate human clinical surgical scenarios. Overall, our study provides an upgraded intraoperative therapeutic platform for localized, tumor-targeted drug delivery in GBM management.

## Materials and Methods

### Material

NH_2_-MSNs (90 nm) were produced from So-Fe Biomedicine, Inc. (Shanghai, China). Doxorubicin hydrochloride (with purity 98%), chitosan (deacetylated 80%, molecular weight = 500,000), and β-glycerol phosphate disodium salt were purchased from Macklin Biochemistry Tech Inc. (Shanghai, China). Dulbecco’s modified Eagle’s complete medium (DMEM), penicillin and streptomycin, phosphate-buffered saline (PBS), and fetal bovine serum (FBS) were purchased from Procell Life Science & Technology Co., Ltd. (Wuhan, Hubei, China). Cell Counting Kit-8 (CCK-8) was obtained from Dojindo Laboratories (Kumamoto, Japan). Sulfo-Cy5.5-NHS was produced by Acmec Biochemical Co., Ltd. (Shanghai, China). DiO (3,3′-dioctadecyloxacarbocyanine perchlorate) cell membrane staining kit and D-luciferin potassium salt were purchased from Beyotime Biotech Inc. (Shanghai, China). Anti-CD9 (Abcam, Ab236630), anti-HSP70 (Abcam, Ab181606), anti-CD81 (Proteintech, 66866-1-Ig), anti-IBA1 (10904-1-AP), and anti-GFAP (60190-1-lg) were purchased from Proteintech (Wuhan, Hubei, China) and Abcam Company. Cy3-conjugated anti-rabbit IgG antibody (AB0133) and AF647-conjugated anti-mouse antibody (AB0162) were purchased from Abways company (China). Mouse tumor necrosis factor alpha (product no. MM-0132M1), High Mobility Group Protein B1 (product no. MM-44107M1), and interleukin 1 beta (product no. MM-0040M1) enzyme-linked immunosorbent assay (ELISA) kits were purchased from MEIMIAN company (Jiangsu, China).

### Cell line and animals

The U87-luc-mCherry cell line was generously given by Prof. Weiping Gao’s Lab, the GL261-luc cell line was purchased from BNCC (BeNa culture collection), and the mouse astrocyte and microglia were purchased from Procell Life Science & Technology. U87-luc-mCherry and GL261-luc were cultured in complete DMEM, and the mouse astrocyte and microglia were cultured in a cell-specified culture medium. All culture media were provided by Procell. All the cells were cultured at 37 °C in a 5% CO_2_ humidified incubator. All media contained 100 U/ml penicillin and streptomycin and 10% FBS (v/v). Six- to eight-week-old female BALB/c nude and C57BL/6J mice were purchased from HFK Bio-tech Co. Ltd. (Beijing, China). All the animal operations were completed in a special pathogen-free environment under the approval of the Institutional Animal Care and Use Committee at Tsinghua University (Animal Protocol number 20WXM-2).

### Exosome isolation and characterization

Tumor-derived exosomes were purified using differential ultracentrifugation. The nanoparticle tracking analysis (NTA), transmission electron microscopy (TEM), and Western blotting were applied to detect the size, morphology, and biological markers of isolated exosomes. The U87-luc-mCherry and GL261-luc cells were cultured in DMEM without FBS for 48 h. Cell culture medium was collected and centrifuged at 1,000 *g* for 15 min and 10,000 *g* for 15 min to remove cell debris. The exosomes were isolated by centrifuging at 100,000 *g* for 90 min at 4 °C. Exosomes were washed with PBS and recollected by centrifugation at 100,000 *g* for 90 min at 4 °C. The exosomes were resuspended with PBS and stained by following the manual of the DiO cell membrane green fluorescence staining kit (Beyotime, China), and then the stained exosomes were washed with PBS and the exosomes pelleted by centrifugation at 100,000 *g* for 90 min. The DiO-stained exosomes were resuspended with PBS and stored at −80 °C after aliquoting. The size distribution and concentration of exosomes were measured using NTA (Malvern NanoSight, UK) after diluting 500 times with PBS. The markers of exosomes were detected by immunoblotting. CD9 and HSP70 were considered markers of exosomes. The morphology of exosomes was observed under the TEM (Tecnai Spirit, Netherlands). Before observation, the sample was negatively stained with 1% uranyl acetate.

### Exo-DMSNs synthesis and characterization

The fluorescence-labeled NH_2_-MSNs were prepared by the NHS-NH_2_ coupling reaction. One milligram of NH_2_-MSNs was mixed with 1 ml 0.1 M sodium bicarbonate water solution (pH at approximately 8.3 to 8.4) with 300 μg/ml sulfo-Cy5.5-NHS and stirred for 6 h at room temperature. The fluorescence-labeled nanoparticles were collected by centrifugation at 13,000 rpm for 10 min and washed with ultrapure water twice to remove the excess dye. The Cy5.5-labeled DMSNs were produced by adding 1 mg/ml DOX water solution into Cy5.5-labeled MSNs and stirring overnight at room temperature. The DMSNs were collected by centrifugation at 13,000 rpm for 10 min and washed with ultrapure water twice to remove the excess DOX. The Exo-DMSNs were produced by adding exosomes into the DMSNs solution and by sonication (Sonics Vibra-Cell, USA) at 40% strength on ice for 5 min. The Exo-DMSNs were collected by centrifugation at 13,000 rpm for 10 min. All the operations were conducted in a light-protected environment. The hydrodynamic diameter of the particles was measured by dynamic light scattering (Wyatt, USA). The absorbance spectrum was acquired using a plate reader (Perkin Elmer). The in vitro and intracellular colocalization of the DOX, fluorescence-labeled MSNs, and exosomes in Exo-DMSNs were confirmed by the LSM980 confocal microscope (Zeiss, Germany). The colocalization of Exo-DMSNs in U87 was observed after incubating nanoparticles with the cells for 12 h. The DiO fluorescence was detected at an excitation wavelength of 458 nm and an emission range of 481 to 508 nm. MSNs (Cy5.5) were detected at an excitation wavelength of 708 nm and an emission range of 656 to 759 nm. DOX was detected at an excitation wavelength of 514 nm and an emission range of 656 to 759 nm.

The morphology of DMSNs and Exo-DMSNs was observed under the TEM (Tecnai Spirit). Before observation, the samples were negatively stained with 1% uranyl acetate. The exosomes sheathed on MSNs can be confirmed by detecting exosomal markers with Western blotting. The final drug loading was calculated by the drug amount in the supernatant of each wash measured by a plate reader (Perkin Elmer, USA) with 480-nm absorbance. The in vitro drug-release profiles of DMSNs and Exo-DMSNs were determined by measuring the absorbance of DOX in the supernatant. The DMSNs and Exo-DMSNs were incubated in PBS (pH at 7.5 and 6.5) at 37 °C for 7 d. At each time point, the nanoparticles were collected by centrifugation at 13,000 rpm for 10 min and the concentration of the released DOX was quantified to calculate the cumulative release. The zeta potentials of exosomes, MSNs, DMSNs, and Exo-DMSNs were measured using a Zetasizer (Malvern Nano ZSE, UK).

### Exo-DMSNs@CS synthesis and characterization

The chitosan powder was added to 0.5% acetic acid (v/v) water solution and then stirred at room temperature overnight to make 2% chitosan solution (w/v). The β-glycerol phosphate disodium salt powder (β-GP) was dissolved into ultrapure water in a 1:1 ratio (w/w). The β-GP solution was dropped one by one into the stirred 2% chitosan solution in a 1:5 ratio (v/v) on ice. After stirring for 5 min, exosomes, DOX, DMSNs, and Exo-DMSNs solution were added into the CS-GP solution and stirred for another 5 min. Then, the gelation occurred after incubation in a 37 °C water bath for 5 min. The gelation was confirmed by reversing the sample bottle. The hydrogel was frozen at −80 °C overnight and directly freeze dried overnight. The morphology of the freeze-dried hydrogel was observed under a scanning electron microscope. Thermosensitivity and mechanical properties were measured using a rheometer (MCR301; Anton Paar GmbH, Graz, Austria). The storage modulus (*G*′) and loss modulus (*G*′′) were recorded under 25 to 45 °C at a heating rate of 1.5 °C/min. The viscosity of the gel-form hydrogel was measured over a frequency range of 0.1 to 1 Hz at 1% strain.

### In vitro cancer cell targeting evaluation of glioma-derived exosomes

The GL261-luc, mouse astrocyte, and microglia were seeded in a confocal dish (Biosharp, China), and DiO-labeled exosomes were incubated with different cells for 12 h. The cells were fixed with 4% paraformaldehyde for 10 min at room temperature. The cell nucleus was stained with 4,6-diamidino-2-phenylindole (DAPI) (Biosharp). The exosome signal was detected by the LSM980 confocal microscope (Zeiss, Germany) at an excitation wavelength of 489 nm and an emission range of 490 to 506 nm for DiO and at an excitation wavelength of 353 nm and an emission range of 411 to 597 nm for DAPI. Z-Stack was applied during imaging. The intracellular signal was quantified using the Imaris 9 software.

### In vitro antitumor efficacy and biological safety

To assess the in vitro antitumor efficacy of Exo-DMSNs, 5,000 U87-luc-mCherry cells were seeded in 96-well plates per well. Certain DMSNs and Exo-DMSNs were added into the cell medium to make the final DOX concentration of 0, 0.5, 5, and 50 ng/ml and incubated with cells for 24 h. Cell viability was measured using the CCK-8 assay. To evaluate the in vitro biological safety of MSNs, the MSNs and Exo-MSNs were added into the medium to make the final particle concentration of 0, 3.9, 7.8, 15.6, 31.2, 62.5, 125, and 250 μg/ml. Cell viability was measured by following the CCK-8 manual.

### Nude mice orthotropic GBM resection model

The BALB/c nude mice were anesthetized by intraperitoneal injection of avertin (250 to 350 mg/kg), and their heads were fixed in the stereotactic instrument. A 2-cm incision was made with scissors, then a hole located 2 mm posterior and 2 mm lateral to the bregma was drilled with a cranial drill. U87-luc-mCherry cells (5 × 10^5^) were injected into the brain 2 mm deep from the surface of the skull with a Hamilton needle (Hamilton, USA). The tumor burden was determined by bioluminescence.

The tumor was resected on day 7 after the cell implantation. First, the mice were anesthetized by intraperitoneal injection of avertin (250 to 350 mg/kg body weight), and their heads were fixed in the stereotactic instrument. A 2-cm incision was made with scissors on the previous scar, and a 3-mm-diameter cranial window with the bone hole as the center was made with a cranial drill. The visible GBM was exposed and removed through an aspirator (Folee Medical, China). A gelatin sponge was used to stop the bleeding, and then the skin was sutured by a 4-0 suture. All resection operations were finished under a stereoscopic microscope (AoMei Technology, China). After the surgery, the mice were intraperitoneally injected with dexamethasone (5 mg/kg body weight) to mitigate the edema.

### Bioluminescence imaging

Bioluminescence imaging with the IVIS Spectrum system (PerkinElmer) was conducted to monitor the relative size of the intracranial GBM. The mice were anesthetized with 2% isoflurane and placed on the imaging plate after intraperitoneally injecting D-luciferin (150 mg/kg body weight) for 10 min, and then the imaging was conducted with auto exposure. The total photon flux per second around the brain area was considered as the tumor burden. The analysis was performed using Living Image software.

### Inhibition of GBM recurrence by DOX@CS and Exo-DMSNs@CS

For DOX@CS, after orthotopic GBM resection, the mice were randomly divided into 3 groups (*n* = 3 per group): group 1, no surgery; group 2, surgery only; and group 3, DOX@CS (5 μg DOX/mouse). GBM resection was not conducted on the mice in group 1. The mice in group 2 accepted GBM resection only. The DOX@CS was dropped into the resection cavity of the mice of group 3. The relapse was monitored by IVIS, and the survival time was recorded. To further study the effect of exosomes on the drug targeting, the mice were randomly divided into 4 groups (*n* = 6 per group): group 1, surgery only; group 2, CS; group 3, chitosan-β-glycerol phosphate hydrogel containing doxorubicin-loaded mesoporous silica nanoparticles (DMSNs@CS); and group 4, chitosan-β-glycerol phosphate hydrogel containing doxorubicin-loaded mesoporous silica nanoparticles encapsulated within tumor-derived exosomes (Exo-DMSNs@CS). The mice in group 1 accepted GBM resection only, and the CS, DMSNs@CS, and Exo-DMSNs@CS were dropped into the resection cavity of mice in groups 2, 3, and 4, respectively; the dose of DOX for each mouse was 7.5 μg. The tumor burden was measured with IVIS every 5 d for 20 d after treatment. The mice were observed every day for survival following for 55 d after treatment. To monitor the health condition of mice after surgery, the body weight was measured for 14 d after the treatment.

### In vivo release of Exo-DMSNs and DMSNs from CS

The liquid CS containing Cy5.5-labeled DMSNs and Exo-DMSNs were dropped into the resection cavity during the orthotopic GBM resection procedure. The fluorescence signal of Cy5.5 was captured and analyzed using the IVIS Spectrum system (PerkinElmer) in fluorescence mode with 675 nm excitation and 750 nm emission every 5 d after the hydrogel implantation for 15 d.

### In vivo biological safety of the hydrogel and tumor targeting of Exo-DMSNs

On day 14 after hydrogel implantation, the blood sample was collected through cardiac puncture, and then the main tissues (lung, liver, kidney, heart, and spleen) and tumor-bearing brain were taken after the mice were euthanized through perfusion. The tissues underwent cryosection and hematoxylin and eosin (H&E) staining. To evaluate the biological safety, the whole slide with H&E staining was scanned by the Pannoramic SCAN system (3DHISTECH, Hungary). To detect the signal of Exo-DMSNs and DMSNs, the slide was observed under the LSM980 confocal microscope (Zeiss) after staining the nucleus with DAPI. Part of the collected blood samples was used to conduct a hematological examination using a hematology analyzer. The serum was obtained from the supernatant of the blood samples after centrifugation at 3,000 rpm for 10 min. The biochemistry indicators of serum were measured using an automatic biochemical analyzer. For the long-term biosafety evaluation, inflammation and behaviors were evaluated via density of brain microglia with immunofluorescence staining (IBA1-positive stained cells), systemic inflammatory cytokine levels with serum ELISA, and cognitive function with Morris water maze. Behavioral testing was conducted under reversed light–dark cycles to minimize circadian interference. All surgical procedures and postoperative care followed the same protocol used for the orthotopic glioblastoma resection model. Briefly, 6- to 8-week-old female C57BL/6J mice were anesthetized and a cranial window was made. A small volume of brain tissue was removed using an aspirator. Hemostasis was achieved with a gelatin sponge, after which the same volume of hydrogel used in therapeutic studies was implanted into the cavity. The long-term biosafety assay was conducted on day 30 postimplantation. The Morris water maze assay was performed as previously described [25]. Briefly, the assay was conducted in a circular water pool (120 cm diameter) filled with white-stained water maintained at 23 to 25 °C. The water pool was divided into 4 quadrants from the center of the pool. A hidden platform was submerged in the center of one quadrant. During each trial, mice were placed on the edge of the pool in each quadrant to allow them to swim freely until they found the platform. Each mouse performed 4 trials per day (one from each quadrant) over 5 consecutive days. The swimming paths were recorded with a camera, and the total traveling distance was analyzed using Ethovision XT software (version 11.5). For immunofluorescence and serum ELISA, the blood was collected through cardiac puncture and brain tissues were isolated after sacrifice by perfusion. Serum was obtained from the supernatant of the blood sample by centrifugation at 3,000 rpm for 10 min. The inflammatory factors (tumor necrosis factor alpha, High Mobility Group Protein B1, and interleukin 1 beta) in serum were quantified using commercial mouse ELISA kits according to the manufacturer’s instructions. The absorbance was measured with a plate reader (Perkin Elmer, USA) at 450 nm. For immunofluorescence staining, brain tissues were fixed, paraffin embedded, and sectioned. The paraffin sections were stained with primary antibodies against ionized calcium-binding adapter molecule 1 (IBA1) and glial fibrillary acidic protein (GFAP), followed by the appropriate fluorescently tagged secondary antibodies. The slides were scanned using an Axio Scan Z1 scanner (Zeiss). The signal densities of IBA1 and GFAP were calculated using ImageJ software.

### Statistical analysis

Data are shown as the standard error or standard deviation of the mean, and a *t* test was conducted for the comparison of antirelapse efficacy. The value of *P* <0.05 was considered statistically significant. Statistical analysis and graphing were performed using GraphPad Prism 9.0 software.

## Results

### Characterization of tumor-derived exosomes and cancer cell targeting

**Fig. 2.**
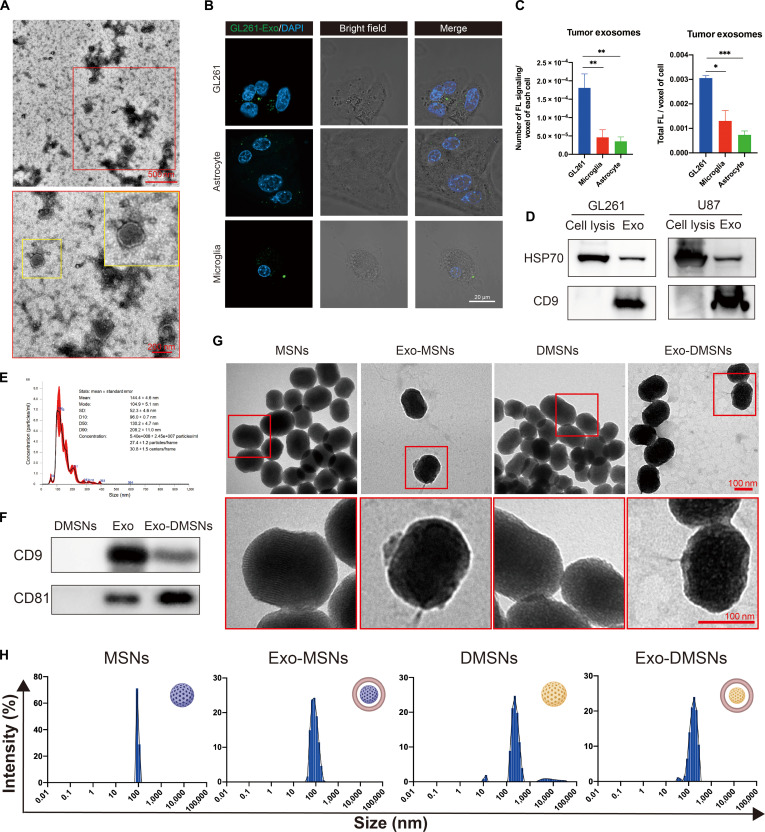
Fabrication and characterization of Exo-DMSNs. (A) Morphology of isolated tumor-derived exosomes. (B) Confocal imaging of intracellular 3,3′-dioctadecyloxacarbocyanine perchlorate (DiO)-labeled exosomes in GL261, astrocyte, and microglia. DAPI, 4,6-diamidino-2-phenylindole. (C) Tumor-derived exosomes’ fluorescence signal of GL261-Exo in GL261, astrocyte, and microglia. *n* = 3 for each group; data are presented as mean ± standard error of the mean (s.e.m.). *P < 0.05, **P < 0.01, and ***P < 0.001. FL, fluorescence. (D) Western blotting for detecting exosomal marker expression in the exosome sample. (E) Size distribution and concentration of isolated exosomes measured by nanoparticle tracking analysis. (F) The exosomal markers on the MSNs detected by Western blotting. (G) The morphology of MSNs, Exo-MSNs, DMSNs, and Exo-DMSNs observed under a transmission electron microscope. (H) The size distribution of MSNs, Exo-MSNs, DMSNs, and Exo-DMSNs measured by dynamic light scattering.

The tumor-derived exosomes were isolated from the cell culture medium of human GBM cell line U87 and mouse GBM cell line GL261 through differential centrifugation. The membrane structure of negatively stained exosomes was observed under the TEM. The spherical morphology and the diameter of exosomes (Fig. 2A) match the previous report [12]. The concentration and size distribution of exosomes were measured by NTA. The mean value of the nanoparticle size of 144 ± 4.6 nm matches the TEM result, and the distribution of particle size was gathered at 100 to 200 nm (Fig. 2E). After diluting the exosomes 500 times with PBS, the concentration of particles was 5.4 × 10^8^ particles/ml, indicating that highly concentrated exosomes were successfully isolated. To further confirm that the isolated membrane structure was exosomes, the markers of exosomes heat shock 70 kDa protein (HSP70) and Cluster of Differentiation 9 (CD9) were detected using Western blotting. The band of exosomal marker CD9 appeared on the exosome sample instead of the whole cell lysis sample. HSP70, which is expressed in both cell lysis and exosomes, is exhibited in both samples. All the results demonstrate that the tumor-derived exosomes were successfully isolated (Fig. 2D). To evaluate the in vitro tumor-targeting feature of tumor-derived exosomes, the GBM cells and normal brain cells, astrocyte, and microglia were incubated with the same amount of DiO-labeled tumor-derived exosomes. The targeting performance was determined by the intracellular amount of fluorescence signal after incubating exosomes with different brain cells (Fig. 2B). In the analyzed result, the intracellular number of DiO-stained exosomes in mouse GBM cells was significantly higher compared to the normal brain cells (Fig. 2C). This result confirms the in vitro tumor-targeting ability of tumor-derived exosomes in the absence of a tumor microenvironment, which simulates the scenario of residual cells after the tumor resection.

### The fabrication and characterization of the Exo-DMSNs

**Fig. 3.**
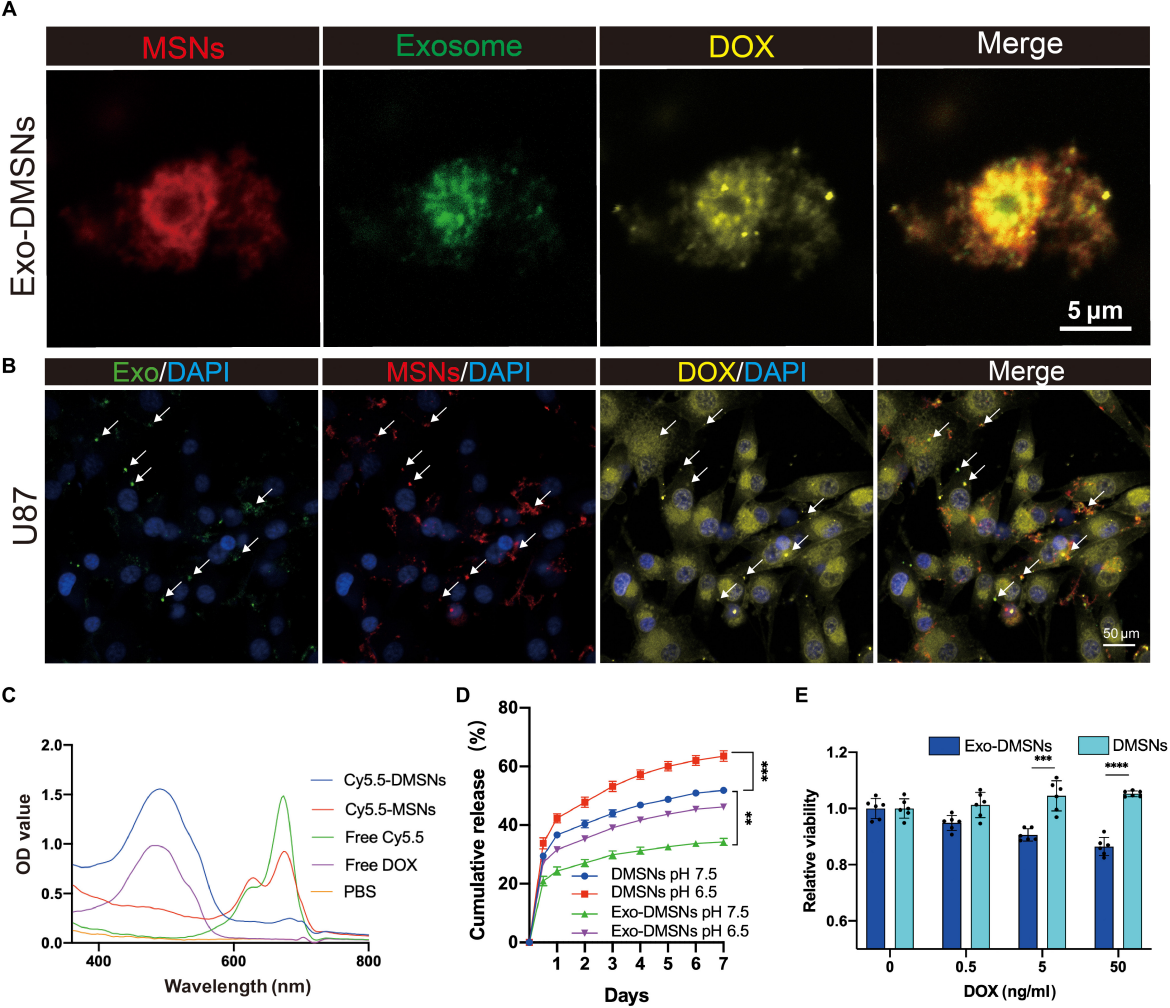
Characteristics of Exo-DMSNs. (A) The in vitro colocalization of the fluorescence signal of Cy5.5, DiO, and DOX autofluorescence. (B) Cellular uptake of Exo-DMSNs. (C) The absorbance spectrum of phosphate-buffered saline (PBS), free DOX, free Cy5.5, Cy5.5-MSNs, and Cy5.5-DMSNs. OD, optical density. (D) The in vitro released DOX profile of DMSNs and Exo-DMSNs in PBS (pH at 7.5 and 6.5). *n* = 3 for each group; data are presented as mean ± SD (***P < 0.001, **P < 0.01). (E) In vitro U87 antitumor efficacy of Exo-DMSNs and DMSNs. *n* = 6 for each group. Data are presented as mean ± SD (***P < 0.001, ****P < 0.0001).

To obtain tumor-targeting drug delivery particles, Trojan-horse-like glioma-cell-derived exosomes based on DOX-releasing MSNs were constructed. To precisely detect the in vitro and in vivo location of Exo-DMSNs via fluorescence, the MSNs and exosomes were labeled with fluorescent molecules. NH_2_-MSNs were fluorescence labeled by Sulfo-Cy5.5-NHS via the NHS/NH_2_ coupling reaction. DOX was loaded to the fluorescence-labeled NH_2_-MSNs by stirring. The peak around 480 nm of free DOX and Cy5.5-DMSNs in the absorbance spectrum demonstrates that DOX was successfully loaded into the fluorescence-labeled NH_2_-MSNs (Fig. 3C). The green-fluorescence-labeled tumor-derived exosomes were encapsulated into drug-loaded Cy5.5-MSNs via sonication. The morphologies of MSNs, Exo-MSNs, DMSNs, and Exo-DMSNs were observed under the TEM. The successful exosomal encapsulation can be demonstrated via the dark layer of the membrane structure surrounding the MSNs that cannot be found in the MSNs and DMSNs group (Fig. 2G). No morphological difference was found between the DMSNs and MSNs as well as between Exo-MSNs and Exo-DMSNs, indicating that DOX did not affect the exosomal encapsulation and the structure of MSNs. To further confirm the encapsulation of exosomes, the exosomal molecular markers on the Exo-DMSNs were detected by Western blotting, and the colocalization of the fluorescence signal of nanoparticles, DOX, and exosomes was observed under a confocal microscope. The band of exosomal markers CD9 and Cluster of Differentiation 81 (CD81) appeared in the sample of Exo-DMSNs and exosomes instead of MSNs, and the identical location of the fluorescence signal of the 3 components under the laser confocal microscope further confirmed that the exosomes encapsulated DMSNs (Figs. 2F and 3A) and particles can be internalized by the U87 cells (Fig. 3B). Meanwhile, the shift in zeta potential from positive to negative further confirmed the successful exosomal encapsulation (Fig. S1). The particle size of MSNs, Exo-MSNs, DMSNs, and Exo-DMSNs was measured by dynamic light scattering, and the particle size remained within a reasonable range (Fig. 2H). Taken together, the fluorescence-labeled Exo-DMSNs were successfully fabricated for further fabrication of Exo-DMSNs@CS. The drug-release profile from the membrane-coated drug-loaded nanoparticles can be prolonged due to the protective barrier provided by the membrane [16]. The DOX-release profile from MSNs with or without encapsulation was measured over 7 d in solution environments simulating the normal (pH 7.5) and tumor (pH 6.5) tissue microenvironments. The results show that the DOX release rate was significantly decreased with the protection of exosomes at both pH 7.5 and 6.5, which demonstrated that the exosomes can effectively prevent premature drug release from MSNs (Fig. 3D). The DMSNs and Exo-DMSNs were incubated with GBM cell line U87 to evaluate the in vitro enhancement of antitumor efficacy with the assistance of tumor-derived exosomes. The significantly lower cell viability under the treatment of Exo-DMSNs compared to the DMSNs (Fig. 3E) suggests that tumor-derived exosomes enhance antitumor efficacy. Before being applied to the mouse model, the in vitro biological safety of MSNs and Exo-MSNs needs to be evaluated. After incubating with various concentrations of nanoparticles with the U87 cell line for 24 h, 250 μg/ml of MSNs and Exo-MSNs in the culture medium did not show obvious toxicity to the U87 cell line (Fig. S2). Based on the in vitro biosafety of Exo-MSNs and MSNs, the MSNs are suitable for in vivo experiments.

### The fabrication and characterization of the Exo-DMSNs@CS

**Fig. 4.**
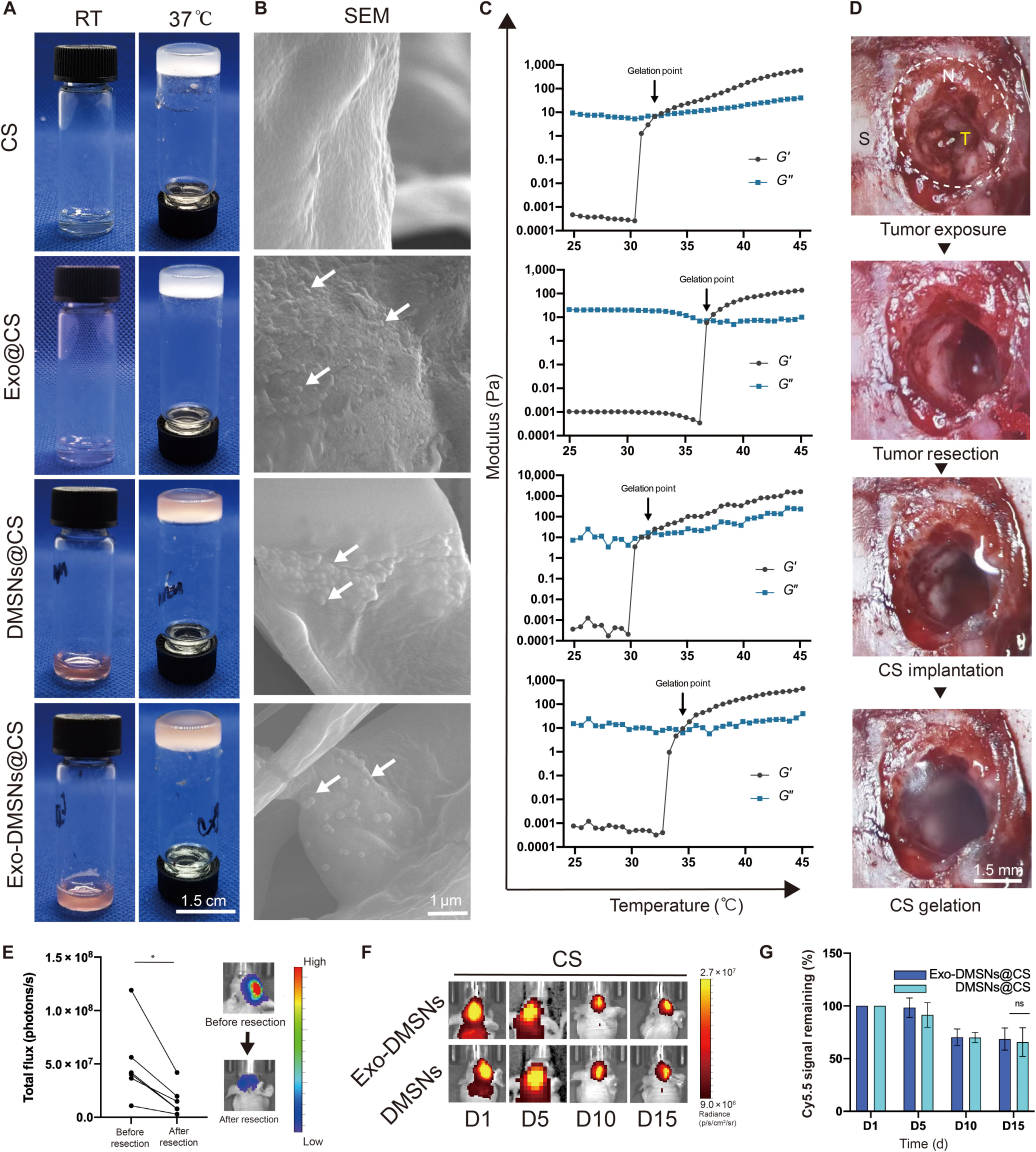
Fabrication and characterization of Exo-DMSNs@CS. (A) The thermosensitive gelation of chitosan hydrogel with/without exosomes, DMSNs, and Exo-DMSNs. (B) The exosomes, DMSNs, and Exo-DMSNs on chitosan observed under a scanning electron microscope (SEM). (C) Physical strength and thermosensitivity of hydrogel. *G*′, storage modulus; *G*″, loss modulus. (D) Procedure of surgery and in situ gelation of CS in tumor cavity. S, skull; N, normal brain tissue; T, tumor tissue. Dashed line indicates the surgery window. (E) Residual tumoral signal after the resection of brain tumor (*P < 0.05). (F) The in vivo release of DMSNs and Exo-DMSNs from CS. (G) The quantitative analysis of the remaining signal of Cy5.5 in the brain area. Data are shown as mean ± SD, *n* = 3 for all groups. ns, no significance.

The Exo-DMSNs@CS was fabricated by loading Exo-DMSNs into the injectable chitosan hydrogel. The thermosensitive chitosan was fabricated by adding β-GP and Exo-DMSNs into the chitosan solution. After 37 °C of heating in a water bath for 5 min, the CS-GP was transferred to a translucent hydrogel from a transparent liquid. The gelated hydrogel maintained on the bottom of the sample bottle showed considerable solid-like stability (Fig. 4A). To ensure that the mechanical properties of the hydrogel were suitable to apply to the brain, the thermosensitive mechanical properties of the hydrogel were measured using a rheometer. The *G*′ curve that increased and crossed with the *G*′′ curve at 30 to 37 °C demonstrated the thermosensitive properties of the Exo-DMSNs@CS system (Fig. 4C). At 37 °C, no shear happens in the frequency sweep test, showing a qualified mechanical strength and stability of the hydrogel (Fig. S3). Meanwhile, the mechanical properties of the hydrogel matched with the stiffness of the brain tissue [26]. The intraoperative thermosensitive gelation was tested in the tumoral resection cavity, and the gelation was also induced at the body temperature of mice (Fig. 4D). To confirm that the nanoparticles were loaded into the hydrogel, the morphologies of CS, Exo@CS, DMSNs@CS, and Exo-DMSNs@CS were observed under a scanning electron microscope. Distinct nanoparticles with reasonable size found on the surface of the hydrogel (white arrow) showed that the nanoparticles were successfully loaded into the hydrogel (Fig. 4B). To evaluate the in vivo release of nanoparticles from the hydrogel, the controlled release was determined by the remaining Cy5.5 signals in the brain area. The result showed that the DMSNs and Exo-DMSNs could be controlled released for more than 15 d. No significant alternation was found in the speed of release between Exo-DMSNs and DMSNs (Fig. 4F and G). Furthermore, obviously concentrated Cy5.5 signals in the brain area demonstrated that the Exo-DMSNs@CS maintains a relatively high local nanoparticle concentration and it may decrease relative to the drug concentration in systemic circulation. However, drug distribution should be evaluated by absolute quantitative measurement of drug concentration in further research to confirm the advantage of local drug delivery.

### Tumor-derived exosomes enhance the inhibition of GBM relapse

**Fig. 5.**
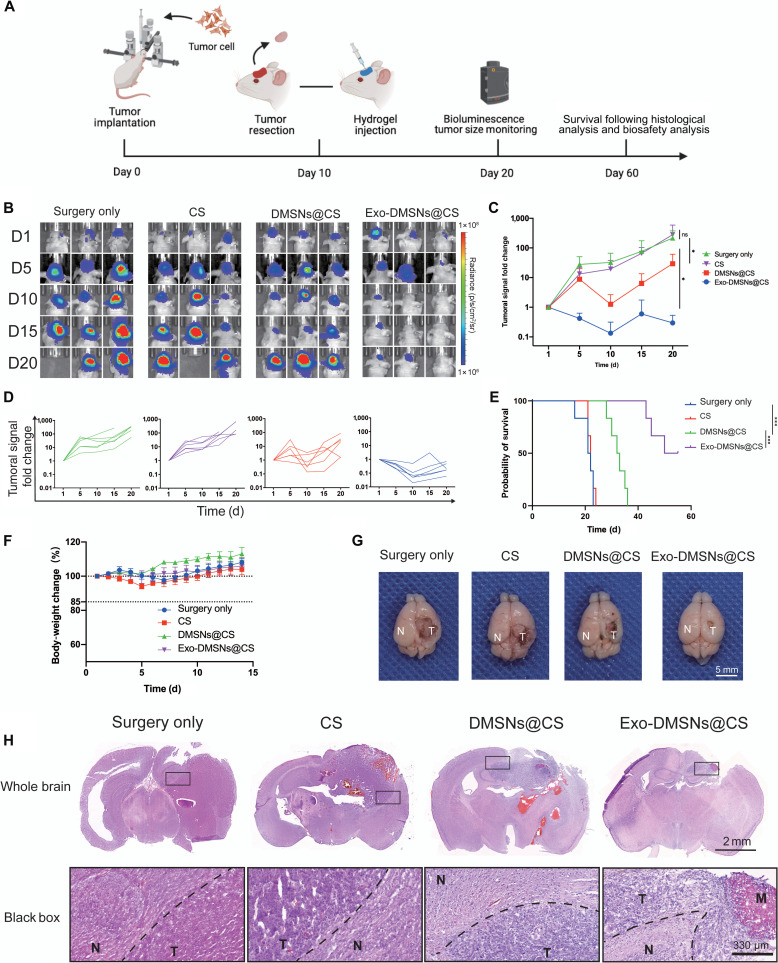
In vivo therapeutic efficacy of Exo-DMSNs@CS. (A) Schematic diagram of the antirelapse experiment. (B) In vivo bioluminescence imaging of relapsed GBM after the surgery and implanting of the CS, DMSNs@CS, and Exo-DMSNs@CS. (C) The tumoral signal fold change of bioluminescence quantification in (B). Data are shown as mean ± s.e.m.; ns, no significance, *P < 0.05. (D) The tumoral signal fold change of individual mouse. *n* = 6 for each group. (E) Cumulative survival of mice after surgery and CS hydrogel treatment. Statistical significance was calculated using a log-rank test. *n* = 6 per group, ***P < 0.001. (F) The body-weight changes after surgery and CS hydrogel treatment. The dotted line indicates 100% and 85%. (G) Tumor-bearing brain collected from each group for 2 weeks after the treatment. N, normal brain tissue; T, tumor tissue. (H) Hematoxylin and eosin staining of the tumor-bearing brain in each group for 2 weeks after the treatment. N, normal brain tissue; T, tumor tissue; M, hydrogel. The dashed line indicates the margin of the tumor.

**Fig. 6.**
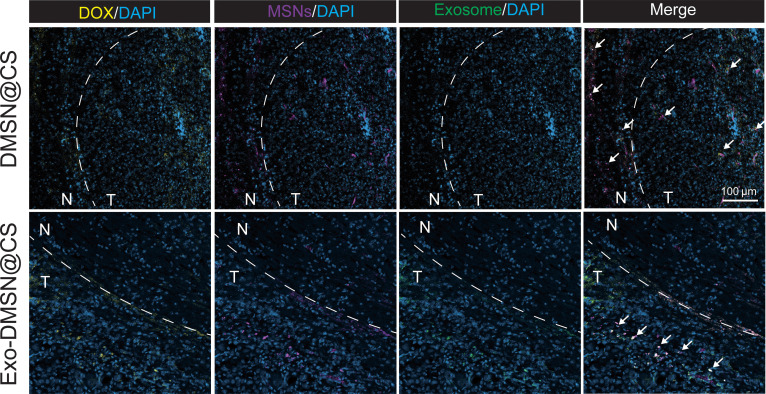
In vivo Exo-DMSNs tumor cell targeting. The dashed line indicates the margin of tumor and normal brain tissue. N, normal brain tissue; T, tumor. The signals of nanoparticles are pointed out by the white arrow; scale bar = 100 μm.

DOX loaded in chitosan for intraoperative GBM was only tested in the subcutaneous tumor model instead of in the orthotopic GBM resection model [27,28]. For further evaluation of the in vivo antirecurrent efficacy of DOX and simulating the actual clinical treatment of GBM, the orthotopic GBM resection model was established. After tumor resection, significant decreases in the tumoral signal on the brain area demonstrated the successful establishment of the orthotopic GBM resection model (Fig. 4E). The survival rate and the tumor burden were recorded after the DOX-loaded chitosan was implanted into the tumor resection cavity (Fig. S4A and B). The survival rate of the mice in the resection-only group was significantly higher than the no-resection group, which simulated the actual clinical outcome that the GBM resection can extend the survival rate (Fig. S4B). Comparing the speed of tumor relapse in surgery-only group and DOX@CS group, the slower relapse rate and extended survival rate of mice in the DOX@CS group confirm that the DOX released from the chitosan was helpful for the inhibition of GBM relapse (Fig. S4A and B). Next, we evaluated whether the tumor-derived exosomes further enhance the inhibition of GBM relapse. In the orthotopic GBM resection model, we treated the mice with surgery only, CS, DMSNs@CS, and Exo-DMSNs@CS. The relapse inhibition was assessed via survival following and bioluminescence imaging (Fig. 5A). After following the tumor burden for 20 d, no antitumor efficacy of the chitosan hydrogel can be observed by the same tumoral signal fold change between the surgery-only group and the CS group. Compared to the surgery-only and CS groups, the reduced relapse rate in the DMSNs@CS group showed the partial antitumor efficacy of DMSNs. The tumor relapse rate of mice in the Exo-DMSNs@CS group was significantly lower than that in the other groups (Fig. 5B to D). This indicates that the efficacy of the antirecurrent can be enhanced by the tumor-derived exosomes. The survival result also was consistent with the rate of tumor relapse. Compared to the surgery-only and the CS groups, the survival time of the DMSNs@CS group was extended; furthermore, the Exo-DMSNs@CS group showed the longest survival time (Fig. 5E). Under the same dose of DOX, the tumor-derived exosomes improved the therapeutic efficacy. To roughly evaluate the biosafety, the body weights of each group were recorded for 14 d after the surgery (Fig. 5F). The body weight of mice in all groups did not decrease more than 15%. The alternation of body weight suggested that the toxicity of the drug delivery system was acceptable. For detailed assessment of the interaction between the hydrogel and parenchyma, the morphology of the tumor-bearing brain was observed via photograph and H&E staining (Fig. 5G and H). In the surgery-only group and the CS group of the tumor-bearing brain, the obvious GBM tissue showed that the residual tumor tissue after resection had already recurred. Under the treatment of DOX, tumor tissue with decreased size was found in the DMSNs@CS and Exo-DMSNs@CS groups (Fig. 5G). In the H&E-staining result, the brain was mostly occupied and compressed by a deeper purple-stained GBM tissue in the surgery-only group and the CS group (Fig. 5H). The tumor area shrank in the DMSNs@CS group compared to the surgery-only and CS groups, and the Exo-DMSNs@CS showed the smallest tumor size. The area in red color is DOX in the hydrogel, which means that the hydrogel remains in the brain tissue and perfectly attaches to the tumor tissue (Fig. 5H). To evaluate the in vivo tumor targeting, the fluorescence signal of Exo-DMSNs and DMSNs was found in the margin of normal brain tissue and tumor tissue. The Exo-DMSNs can be found mainly in the tumor tissue side, while DMSNs can be found on both sides (Fig. 6), which suggested the tumor-targeting effect of Exo-DMSNs. Taken together, these results indicate that the Exo-DMSNs@CS contain the best antitumor efficacy within acceptable toxicity compared with other groups.

### Biosafety evaluation

To evaluate systemic toxicity, the histological morphologies of major organs including the kidney, liver, spleen, lung, and heart on day 14 after treatment were evaluated by H&E staining. Also, blood-chemistry analysis was used to evaluate the functional integrity of major organs (Fig. S5A and C). No obvious focus in all slides and significant alterations in the blood-chemistry analysis were found. These results indicated that the function of major organs was not impaired by treatment. Major hematological parameters remain within a reasonable zone in the complete blood count (Fig. S5B). The short-term in vivo biosafety evaluated from immunodeficient mice is insufficient for assessing the long-term safety for brain retention. Therefore, we conducted a 30-d evaluation in healthy immunocompetent mice. The stable growth of body weight after the hydrogel implantation indicated no acute sickness (Fig. S6A). To evaluate potential systemic and regional inflammation, we measured serum cytokine levels and quantified the density of microglia in the brain. The absence of significant changes in inflammatory cytokines (Fig. S6B) and microglial activation confirmed that the treatment did not provoke additional systemic or local inflammatory responses (Fig. S6D and E). To evaluate the impact of the hydrogel on cognitive function, we conducted the Morris water maze assay for spatial learning ability assessment. Comparable path lengths to locate the hidden platform across groups indicated that the delivery system did not impair learning ability (Fig. S6C). Together, the long-term and short-term biosafety results demonstrate an acceptable safety profile for the implanted delivery system.

## Discussion

This study demonstrates an innovative approach to achieve long-lasting, intraoperative tumor-targeted drug delivery for the treatment of glioblastoma through developing a chitosan-based hydrogel delivery system loaded with glioma-derived exosomes encapsulating DOX-loaded MSNs. Our results reveal that controlled release of Exo-DMSNs from thermosensitive hydrogel markedly improves GBM recurrence prevention compared to non-exosomal DMSNs. Our design overcomes the limitation of both the off-target side effects and suboptimal antirecurrence efficacy associated with free drug or conventional drug-loaded nanoparticles. First, glioma-cell-derived exosomes were used to encapsulate DOX-loaded MSNs, enabling localized and precise tumor-targeted drug delivery and thereby enhancing therapeutic efficacy and reducing off-target cytotoxicity. Second, the chitosan delivery system enables the long-term controlled release of therapeutic payloads via adjustable degradation rates and the electrostatic interaction between the negative charge on exosomes and the positive charge on chitosan, thereby addressing the high postoperative relapse rate of glioblastoma. Third, the thermosensitive chitosan-based system loaded with glioma-derived exosomes we developed enables in situ injectable drug delivery and sufficient cavity-filling capability, thus achieving excellent therapeutic efficacy and in vivo biosafety. Collectively, all the results demonstrate the feasibility, effectiveness, and biosafety of the exosome-loaded chitosan-based drug delivery system in the context of intraoperative glioblastoma therapy.

Currently, the pronounced inter- and intratumoral heterogeneity of GBM poses a major challenge for the development of molecular targeted therapies, making chemotherapy remain as a mainstay treatment option for GBM [29]. Although certain approaches, such as immune checkpoint inhibitors and cancer vaccines, have shown promise in preclinical models and early-phase trials, multiple phase III randomized controlled trials have failed to demonstrate marked improvements in overall survival. The clinical efficacy of immunotherapy for glioblastoma remains limited. This is largely attributable to the highly immunosuppressive tumor microenvironment of GBM, characterized by low T-cell infiltration, abundant myeloid-derived suppressor cells, and inhibitory cytokines. Consequently, no immunotherapy has yet been approved by the U.S. Food and Drug Administration specifically for glioblastoma [30], and the combination of chemotherapy with surgery and radiotherapy continues to serve as the clinical mainstay. Thus, the conventional chemotherapy employed in this study provides a greater opportunity for clinical translation. Improving the efficacy of chemotherapy via targeted delivery that simultaneously increases the antitumor efficacy while reducing systemic toxicity may be beneficial for the majority of patients with GBM. Moving forward, personalized targeted drug delivery to recurrent tumors may be achievable by exploiting the innate tumor-homing feature of patient-derived exosomes. The tumor-targeting specificity of tumor-derived exosomes across different cancer cell lines and normal cells has been demonstrated, but not yet in the cancer cells of the same tumor category derived from different patients or with distinct mutations [12]. Although tumor-derived exosomes provide the possibility of personalized therapy, exosomal heterogeneity arising from cell origin, tumor category, and inconsistent quality (e.g., purity and yield) of isolated exosomes may pose barriers to clinical translation. Therefore, the potential impact of intertumoral heterogeneity on the tumor-targeting feature of exosomes warrants further investigation. Furthermore, glioma stem cells (GSCs) are recognized as a major contributor to GBM recurrence [31]. Since human GSCs can be isolated and cultured in vitro, they also offer a viable source for generating GSC-specific exosomes [32]. In this study, we have confirmed the tumor-homing capacity of glioma-derived exosomes. Building on this finding, future work could explore the use of patient-derived GSC exosomes to selectively target corresponding GSCs in orthotopic GBM models established in immunodeficient mice, which may further enhance therapeutic precision and efficacy.

In this study, we further confirmed the tumor-targeting feature of tumor-derived exosomes previously reported in literature. However, the exact molecular mechanisms governing how these tumor-derived exosomes specifically target tumor tissues require more comprehensive investigation. Meanwhile, because of the technical difficulty in precisely monitoring the release patterns of nanoparticles from hydrogels within the brain tissue, the in vivo interactions between exosome-coated nanoparticles and tumor cells remain to be fully elucidated in the following work. A potential mechanism underlying exosome homing may involve interactions between target-cell membrane proteins and exosomal surface proteins [33]. Previous research demonstrated that CD81, a membrane marker of exosomes detected in our isolated exosomes, can bind to GPR56 [34], a membrane protein regulating cell–cell and cell–extracellular matrix interactions. It is proven that GPR56 expressed in GBM tissue is up-regulated compared to normal brain tissue (analyzed TCGA data from gepia.cancer-pku.cn). We therefore hypothesize that GPR56 overexpression increases the probability of binding to exosomal CD81, thereby promoting preferential uptake of exosomes by GBM cells. Nevertheless, the detailed molecular mechanism underlying the tumor-homing ability of tumor-derived exosomes still remains to be fully elucidated. Additionally, although the advantages of applying tumor-derived exosomes for tumor targeting have been extensively reviewed, the potential tumor-promoting function of these tumor-derived exosomes and their biological impact on normal cells should not be ignored [35], even though we have demonstrated their neurological safety and therapeutic efficacy. The mRNA and protein cargo in tumor-derived exosomes can promote tumor progression by altering the function of cancer-associated cells, including endothelial cells and fibroblasts. In this study, during the preparation of the Exo-DMSNs, the encapsulation process involving sonication would effectively reduce the exosomal cargo [36]. In theory, this reduction should weaken the protumor ability of the tumor-derived exosomes; however, this hypothesis requires further research and experimental evidence. Nevertheless, it is still important to avoid contamination of the delivery system with the isolated tumor-derived exosomes lacking therapeutic payload. In this study, taking advantage of the density difference between MSNs and exosomes, the excess tumor-derived exosomes can be easily removed by centrifugation after sonication. More than that, the localized controlled-release delivery approach may also decrease the opportunity for systemic exposure to any potential unknown biological risks.

DOX has been proved to have excellent antitumor efficacy in vitro [37,38], while it is rarely applied in the clinical treatment of GBM due to the BBB restrictions. In laboratory settings, intravenous administration often requires surface modifications to facilitate BBB penetration, which increases nanoparticle complexity and hinders clinical translation [18,39,40]. Local delivery strategies that bypass the BBB could allow a broader range of drugs to be efficiently transported into GBM cells. Moreover, beyond DOX, other drugs currently used in GBM clinical treatment, such as temozolomide, could also be loaded into MSNs, potentially enhancing both translational potential and therapeutic efficacy. Furthermore, although we have carefully evaluated the biosafety of Exo-DMSNs@CS in the rodent neural system through behavioral and inflammatory assessments, further validation in higher-level animal models (e.g., nonhuman primates) is still required prior to clinical translation. While the individual components employed in this study, including chitosan [41–43], MSNs [44], DOX [16,45], and exosomes [46], have been well characterized for biosafety in preclinical and clinical studies, long-term biosafety monitoring over a period of up to 3 months in a large-animal model or clinical trials remains essential to support further clinical translation. In addition, although the Exo-DMSNs@CS showed excellent antitumor efficacy in this study, it did not achieve complete tumor eradication. Therefore, developing more potent hydrogel-based delivery systems with enhanced antitumor activity remains an important direction for improving therapeutic outcomes.

With the advancement of biomaterial science, numerous injectable hydrogel delivery systems have been designed and fabricated for GBM therapy, with most current designs focusing on modulating the immune microenvironment to eliminate the residual tumor cells [47–49]. This trend highlights intraoperative biomaterial-mediated drug delivery as an emerging therapeutic paradigm for glioblastoma [50]. Although challenges remain in bridging laboratory research and clinical application, material-based drug delivery is experiencing renewed interest and is expected to drive further innovation in intraoperative GBM treatment systems.

## Data Availability

The main data supporting the findings of this study are available within the article and its supplementary information. Extra data are available from the corresponding author upon reasonable request.
