# Supplementary material for: Engineering Glioma-Cell-Derived Exosomes as Trojan Horse for Precisely Targeted Chemotherapy of Glioblastoma
Source: Biomater Res. 2026 Jun 2;30:0365. doi: 10.34133/bmr.0365 (PMC13226992; doi:10.34133/bmr.0365)
Supplement: Supplementary 1 — Figs. S1 to S6 [file bmr.0365.f1.docx]

Supplementary Materials


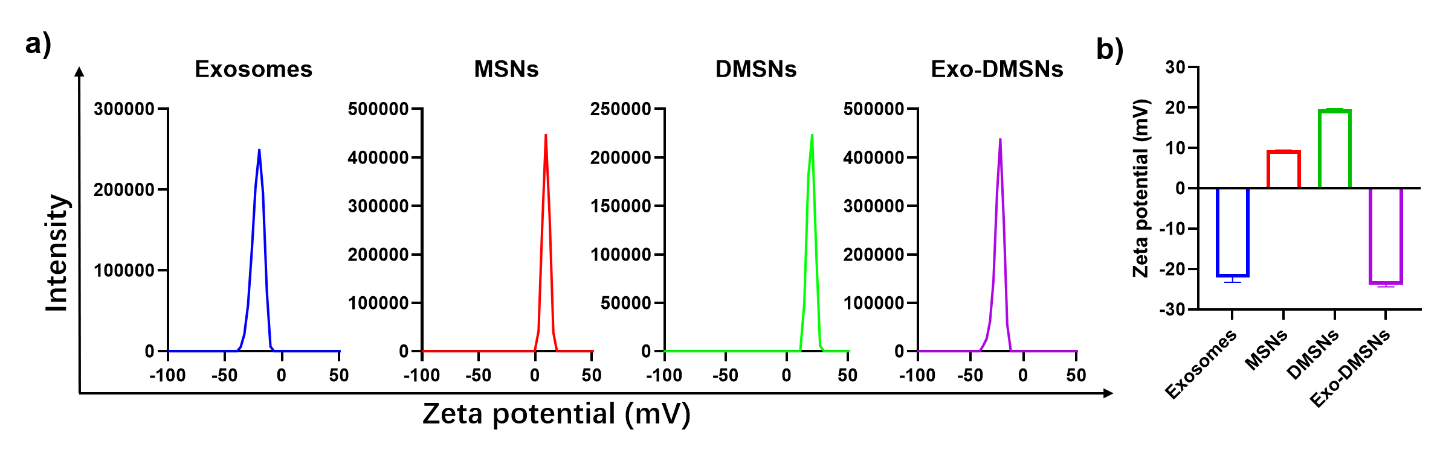


**Figure S1** Zeta potential charge of nanoparticles. a) Distribution of zeta potential of indicated nanoparticles. b) Zeta potential value of indicated nanoparticles. n=3 for each group. Data are shown as mean ± SD.


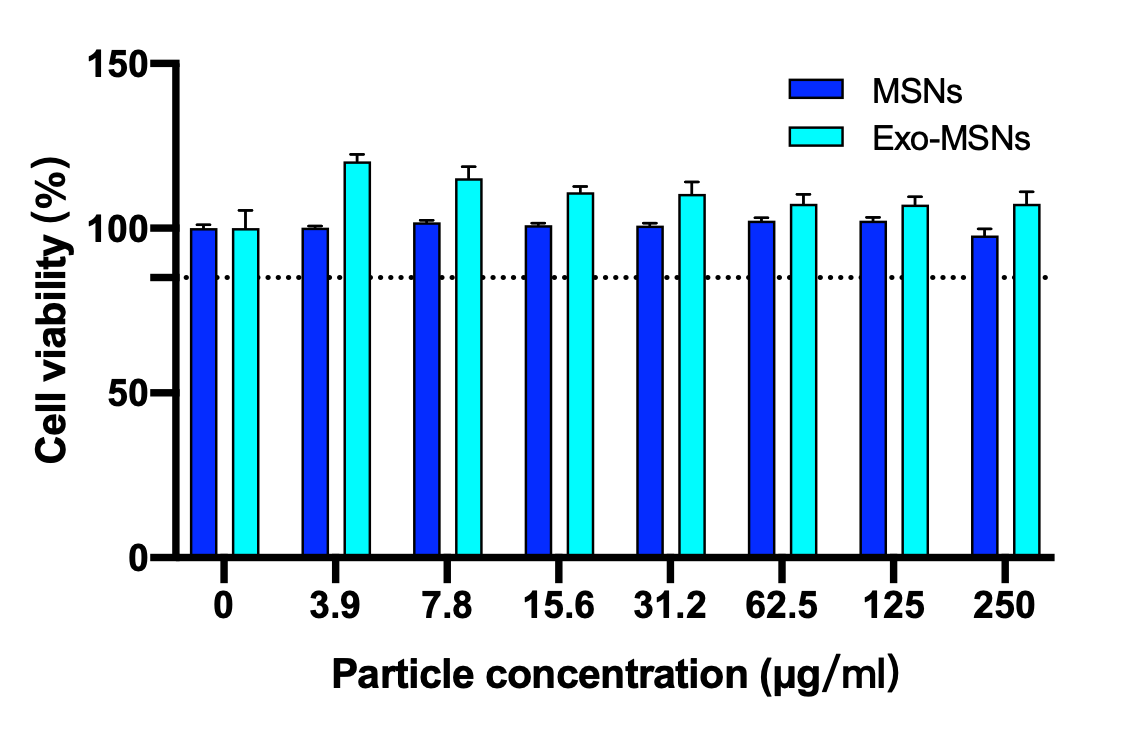


**Figure S2** *In vitro* biosafety of MSNs and Exo-MSNs. Cell viability of U87 was measured by CCK-8 after incubation with MSNs and Exo-MSNs for 24 hours, the dotted line indicates 85%. Data are shown as mean ± s.e.m. (n=6 for each group).


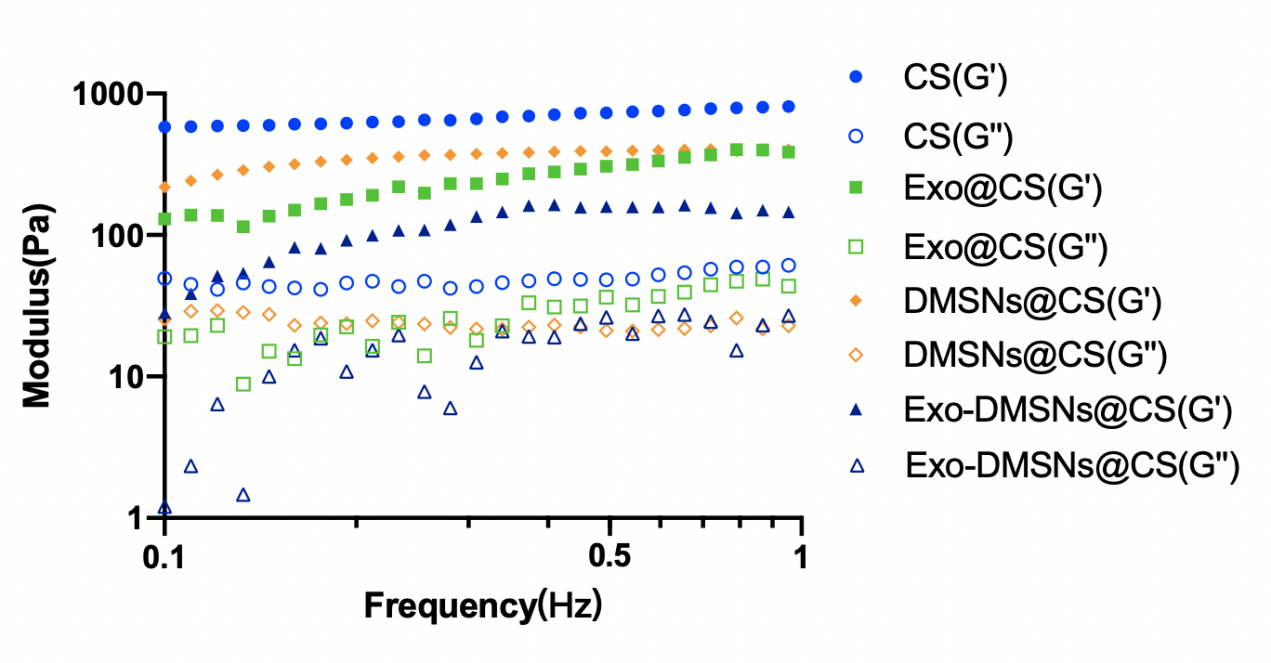


**Figure S3** Dynamic frequency sweep test from 0.1 to 1Hz at 1% increase at 37℃. G’, storage modulus, G’’, loss modulus.


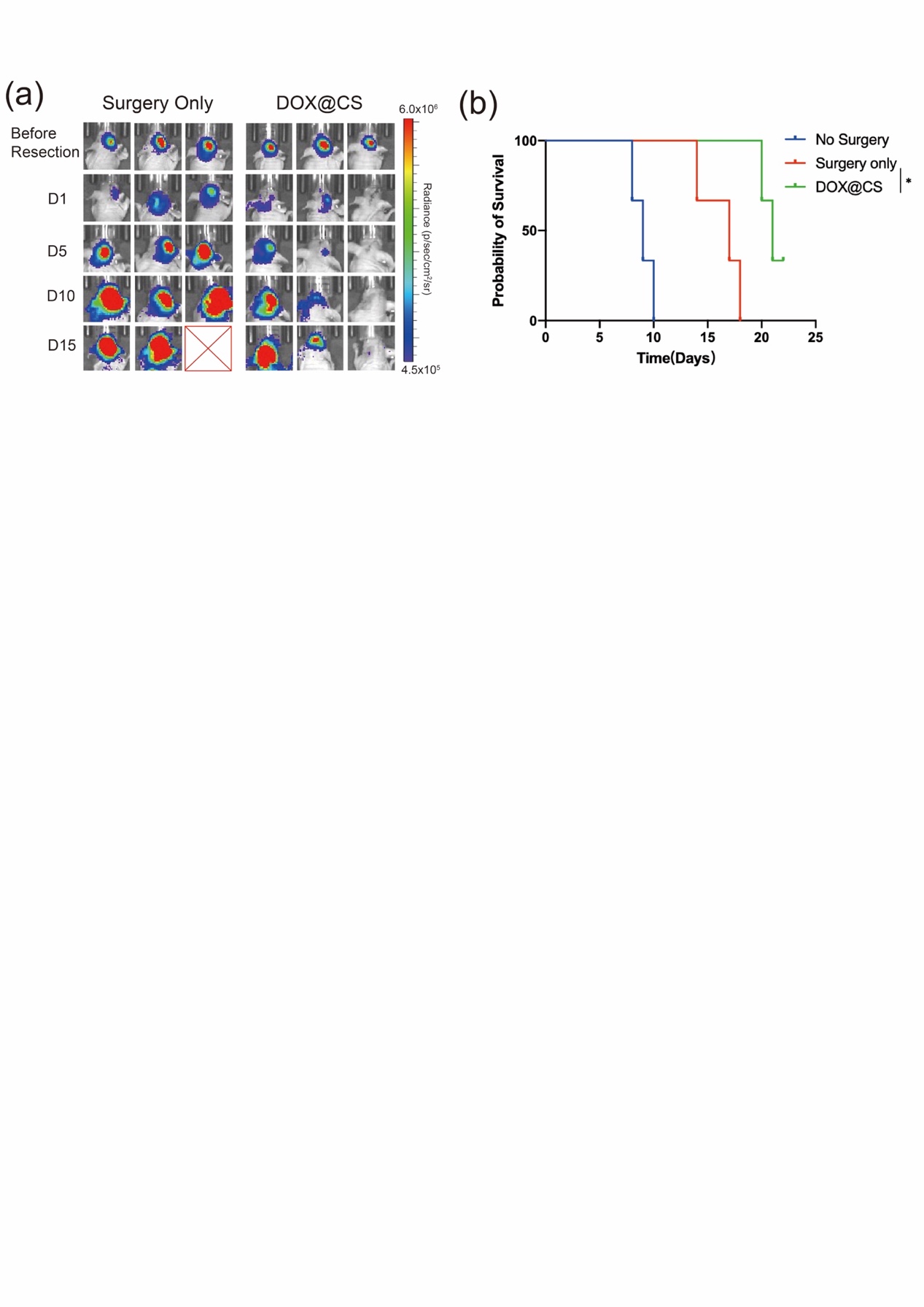


**Figure S4** Anti-relapse efficiency of DOX@CS. a) Tumor relapse speed after tumor resection and DOX@CS implantation. b) Cumulative survival of mice after intracranial tumor implantation. The statistical significance was calculated by Log-rank test. n=3 for all groups, *p<0.05.


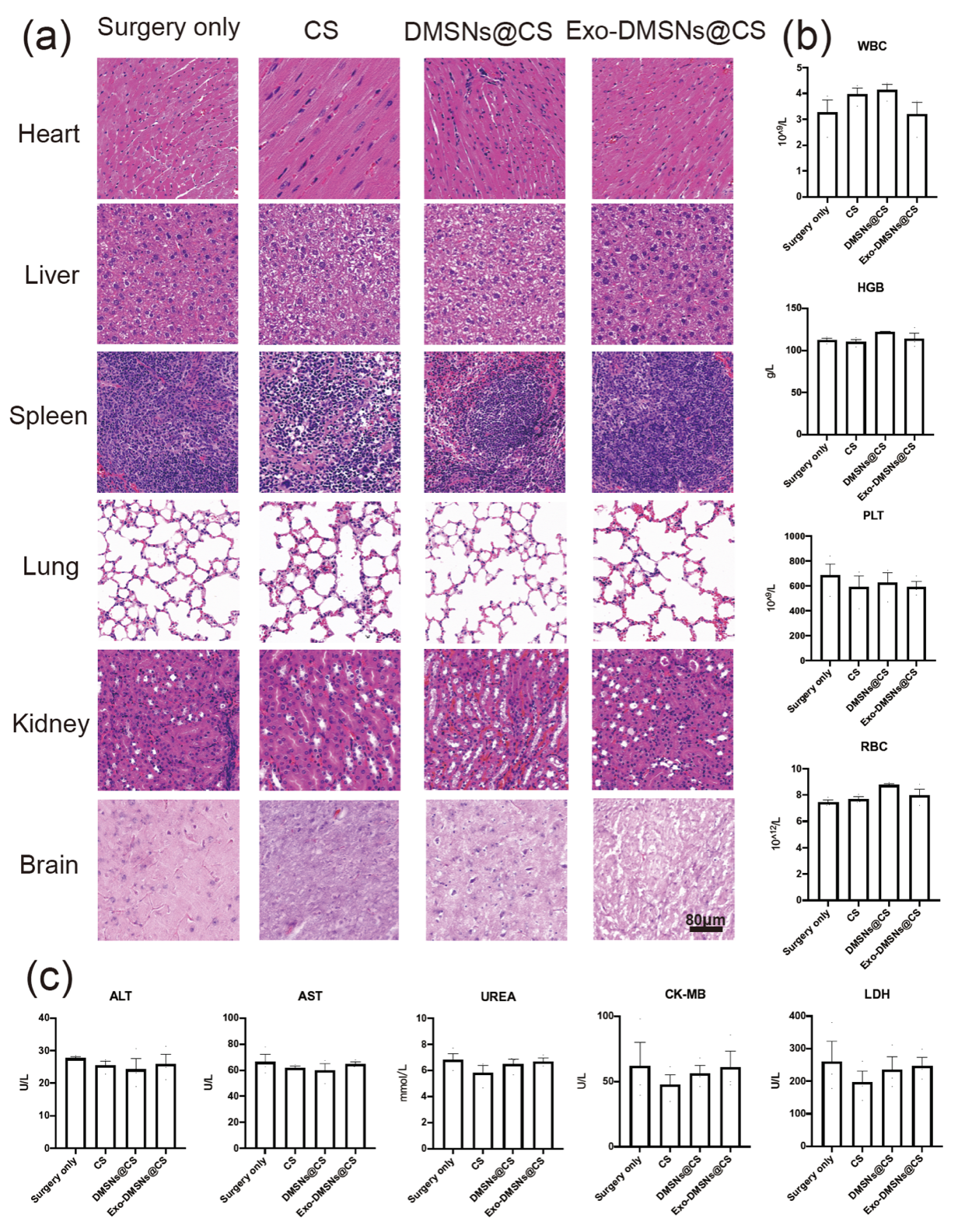


**Figure S5** Biosafety of the delivery system. a) H&E staining of major organ of mice after 14 days of treatment. Scale bar: 80μm. b) Whole blood count of the mice after 14 days of treatment, WBC, white blood cells; HGB, hemoglobin; PLT, platelets; RBC, red blood cells. Data are shown as mean ± s.e.m. (n=3 for each group). c) Blood biochemical test for the mice after 14 days of treatment. ALT, alanine aminotransferase; AST, aspartate aminotransferase; UREA, blood urea nitrogen; CK-MB, creatine kinase isoenzymes; LDH, lactate dehydrogenase. Data are shown as mean ± s.e.m. (n=3 for each group).


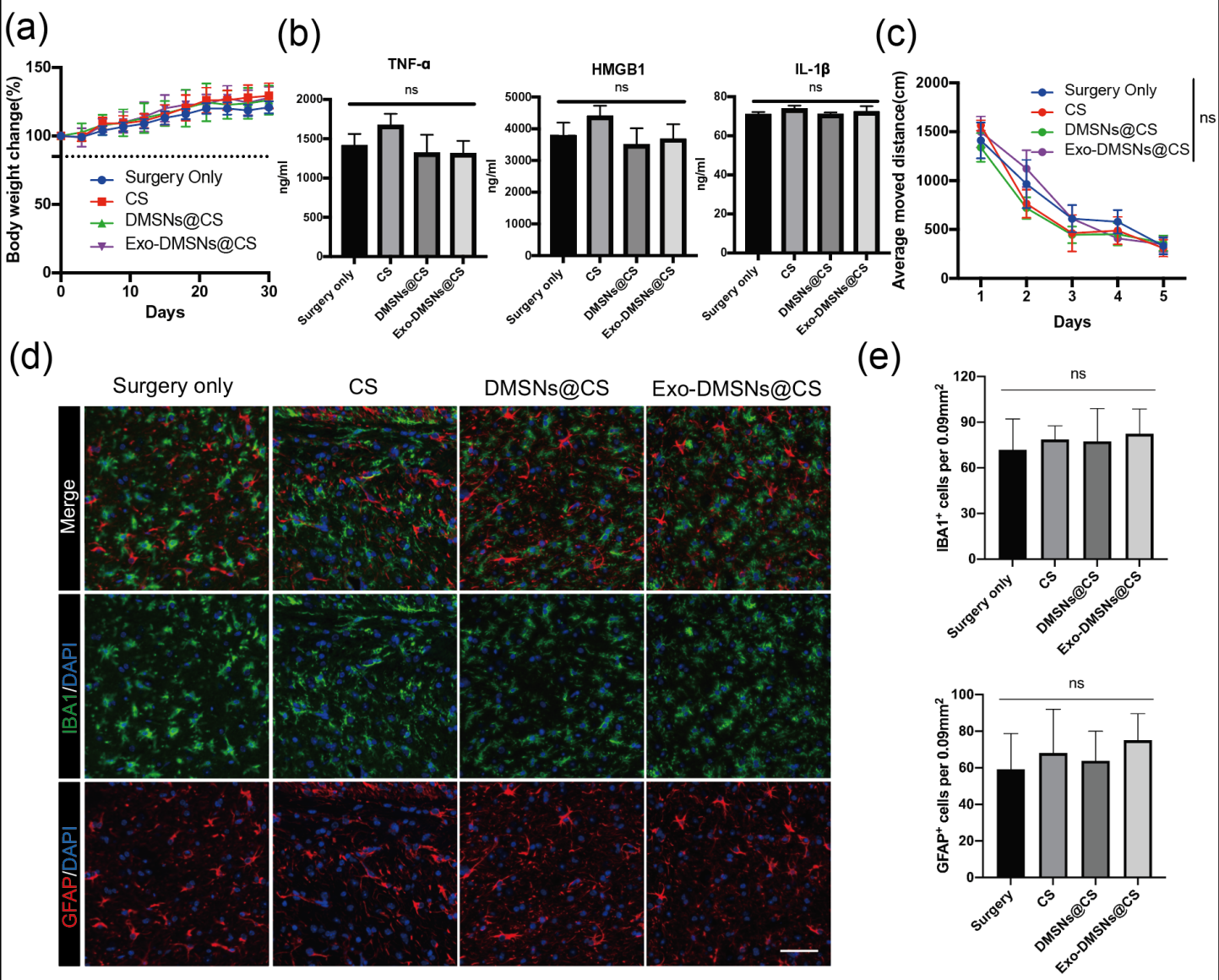


**Figure S6** Inflammation and cognitive evaluation of brain implantation of Exo-DMSNs@CS. a) Mice body weight measurement for 30 days after the implantation of Exo-DMSNs@CS. b) Inflammation cytokine Interleukin 1 beta (IL-1β), Tumor Necrosis Factor alpha (TNF-α), High Mobility Group Protein B1 (HMGB1) concentration in serum determined by ELISA after hydrogel implantation. n=8 for each group. The data are shown as mean ± s.e.m.. ns: no significance. c) Morris Water Maze for cognitive evaluation after the hydrogel implantation. n=6 for each group. The data are shown as mean ± s.e.m.. ns: no significance. d) Microglia and astrocyte defined via Ionized calcium-binding adapter molecule 1 (IBA1) and Glial Fibrillary Acidic Protein (GFAP) signal in immunofluorescence staining located next to hydrogel implant region. Scale bar: 50μm. e) The density of microglia and astrocyte in observed region. n=9 for each group. Data are shown mean ± SD. ns: no significance.
